# Intracranial recording in patients with aphasia using nanomaterial-based flexible electronics: promises and challenges

**DOI:** 10.3762/bjnano.12.27

**Published:** 2021-04-08

**Authors:** Qingchun Wang, Wai Ting Siok

**Affiliations:** 1 Department of Linguistics, The University of Hong Kong, Hong Kong, China

**Keywords:** aphasia, flexible electronics, intracranial electroencephalography (iEEG), language processing, neuroimaging techniques

## Abstract

In recent years, researchers have studied how nanotechnology could enhance neuroimaging techniques. The application of nanomaterial-based flexible electronics has the potential to advance conventional intracranial electroencephalography (iEEG) by utilising brain-compatible soft nanomaterials. The resultant technique has significantly high spatial and temporal resolution, both of which enhance the localisation of brain functions and the mapping of dynamic language processing. This review presents findings on aphasia, an impairment in language and communication, and discusses how different brain imaging techniques, including positron emission tomography, magnetic resonance imaging, and iEEG, have advanced our understanding of the neural networks underlying language and reading processing. We then outline the strengths and weaknesses of iEEG in studying human cognition and the development of intracranial recordings that use brain-compatible flexible electrodes. We close by discussing the potential advantages and challenges of future investigations adopting nanomaterial-based flexible electronics for intracranial recording in patients with aphasia.

## Introduction

Aphasia is an impairment in language and communication, which results from damage to specific brain regions responsible for language [1]. Brain damage can be caused by stroke, tumours, seizures, infection, degeneration, or traumatic brain injury, with stroke being the most common cause of aphasia. According to the US National Aphasia Association, approximately two million members, or 0.6%, of the US population suffer from aphasia, with new cases increasing at a rate of approximately 180,000 per year; also, approximately one third of stroke patients have aphasia. It is projected that by 2030, approximately 4% of US adults will have a stroke [2]. Accordingly, the incidence of aphasia worldwide is expected to increase in the coming decades. Since individuals with aphasia differ greatly in the severity, pattern, and associated lesions of their disorder, it is important to determine the precise location of the lesions and to map the affected brain circuits related to the individuals’ language deficits. The scientific study of aphasia, aphasiology, is important not only because it helps in the formulation of the best treatment methods for restoring the lost cognitive functions of individuals with aphasia but also because it provides another window into the brain mechanisms of language.

Over the last century, researchers from multiple disciplines, including biology, psychology, neuroscience, linguistics, and cognitive science, have attempted to understand the nature of aphasia. The research techniques used include classical post-mortem and histological procedures, and modern neuroimaging methods such as positron emission tomography (PET), magnetic resonance imaging (MRI), functional magnetic resonance imaging (fMRI), diffusion tensor imaging (DTI), electroencephalography (EEG), magnetoencephalography (MEG), and intracranial electroencephalography (iEEG). Each of these methods has pros and cons, and each is used to achieve specific results. MRI and DTI provide in vivo measures of brain anatomy and permit direct examination of how anatomical variations may relate to differences in cognitive functioning. DTI is sensitive to the MR signal of water molecules on a micrometre scale to determine water diffusion in different dimensions in terms of magnitude and direction. DTI offers an opportunity to measure the microstructural characteristics of white matter and allows for the examination of how distinct brain regions are correlated. fMRI and PET extend traditional structural imaging to include maps of human brain function that reveal brain regions involved in the performance of a particular cognitive task. PET and fMRI have good spatial resolution and are useful for localising functional brain activation but have poor temporal resolution. In contrast, EEG has good temporal resolution, but its spatial resolution is rather poor. By placing electrodes on the scalp, EEG detects electrical activations of the brain. EEG has been used rather extensively in clinical settings to diagnose conditions such as epilepsy, brain tumours or damage, stroke, seizures, and sleep disorders by detecting aberrant EEG activity [3]. However, the quality of brain signals recorded from deep subcortical regions, such as the insula and hippocampus, remains questionable [4].

Similar to EEG, iEEG also uses electrodes to detect brain neural activity, but the electrodes are either placed on the surface of the brain or implanted inside the brain. With high temporal and spatial resolution, iEEG is an important tool for localising brain regions of interest [5]. However, because of its invasive nature, iEEG signals can only be recorded in patients for presurgical evaluation and functional mapping, and the electrodes must be implanted for clinical but not research purposes. These rules have restricted the location of electrodes that can be placed and have limited our understanding of more complex neural networks such as language processing that require accurate and precise recording of neuronal activity covering almost the whole brain.

The application of novel nanomaterials has the potential to overcome the limitations of conventional electrode arrays. IEEG electrode arrays electroplated with nanoparticles could lower impedance and allow for a closer contact with cortical cells, thereby providing more accurate recordings of cortical activity [6]. Preclinical tests using animals (rats or primates) have shown that nanomaterial-based electronics could boost the spatiotemporal accuracy and resolution of brain imaging signals [7]. In general, iEEG electrode arrays made of nanomaterials are thinner, lighter, and more flexible and sensitive; these characteristics lead to higher spatial and temporal resolution than that of conventional electrode arrays [6–7]. These features make them less harmful to brain tissue [6], indicating their potential application in the human brain.

In this review, we first introduce classical neuropsychological research on aphasia. We then discuss how non-invasive neuroimaging methods have advanced our understanding of the brain mechanisms of language processing and review the findings of iEEG studies. Finally, we discuss the advantages and limitations of iEEG and discuss how novel nanotechnology may facilitate the study of aphasia.

## Review

### A brief review of classical neuropsychological research on aphasia

The seminal report on aphasia by the French surgeon Paul Broca in 1861 [8] marks the first empirical proof for the functional brain regions of language. Broca observed in his patient, nicknamed Tan*,* that a brain lesion in the ventroposterior portion of the left frontal lobe would lead to speech production difficulties. Tan had intact language comprehension and mental functions but could only produce the sound “tan”. In a later autopsy report of twelve patients with similar symptoms, Broca [9] proposed that the left ventral frontal region was a speech centre for language articulation and production. Broca’s proposal was provocative in his time as the brain was believed to function holistically with no localisation of cognitive functions [10]. Inspired by the proposal of his mentor Theodor Meynert that the left superior temporal gyrus (STG) might be important in speech comprehension, the German physician Carl Wernicke [11] also believed that there were localised brain regions for language functions but argued that Broca’s area was not the only localised speech centre [12]. Wernicke performed an autopsy on a female patient who suffered from comprehension difficulties (she could not understand others’ speech or use the right words for expression) but could speak volubly. He found that the patient had obstruction of the Sylvian artery, and her left STG was damaged. Wernicke termed this type of aphasia *sensory aphasia*, now known as Wernicke’s aphasia, as he believed that the STG (or Wernicke’s area) was a sensory speech area that stored the sound images of object names (or word pronunciations). Destruction of Wernicke’s area leads to difficulty in understanding concepts or word meanings as word pronunciations are lost and could not be used to retrieve word meanings. This loss of word pronunciation also leads to an inability to produce meaningful speech because concepts could not be linked to word pronunciations and could not be translated into motor movements for articulation.

Based on the findings that there were localised regions for speech functions such as motor and sensory speech experience, Wernicke [11] proposed a neurobiological model of language functions that comprises a motor speech centre (i.e., Broca’s area) and a sensory speech centre (i.e., Wernicke’s area), a pathway connecting the two speech centres, an input pathway from auditory nerves to Wernicke’s area, and an output pathway from Broca’s area to motor nerves. He also included memory areas for tactile and optical images, both being parts of the concept or semantic network that do not have localised representations but that are distributed over the whole brain. The tactile memory area (for handwriting) has connections with Broca’s area, whereas the optical memory area (for reading) has connections with both Wernicke’s and Broca’s areas. This information-processing type of model outlines a very preliminary framework of language production and comprehension. It depicts the pathways for listening, speaking, reading and writing, and explains how sensory and motor aphasia, alexia (acquired reading disorders) and agraphia (acquired writing disorders) might be caused. Wernicke also differentiated between disorders caused by a lesion in the speech areas and those caused by damage to the pathway connecting the two speech areas. The former leads to a receptive or expressive problem. The latter leads to conduction aphasia, which is a difficulty in speech repetition with intact comprehension and expression. Although Wernicke did not have empirical evidence to support the existence of conduction aphasia when he proposed the model, his prediction led to the discovery of this rare form of aphasia [12].

**Figure 1:**
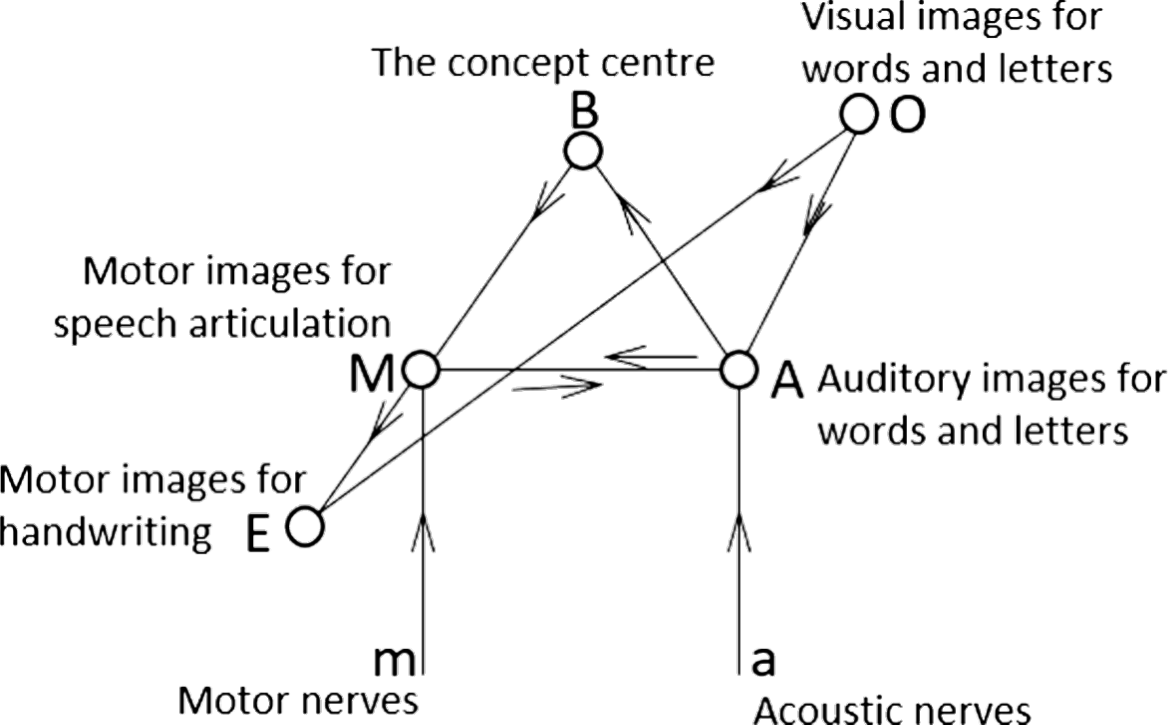
Wernicke’s-Lichtheim model (redrawn from [12], Figure 2). A: The Wernicke’s area. M: The Broca’s area. a: The pathway connecting Broca’s area and the motor nerves. m: The pathway connecting the acoustic nerves and Wernicke’s area.

Wernicke’s model was later found to be inadequate in explaining several special types of aphasia, such as pure word deafness (PWD) and transcortical sensory aphasia (TSA). Patients with PWD cannot understand and repeat speech but can understand written texts and speak normally [12]. TSA has symptoms similar to Wernicke’s aphasia, except that TSA patients can repeat speech normally [12]. To account for these new types of aphasia, Lichtheim [12] modified Wernicke’s model by including a pathway connecting the primary auditory cortex with Wernicke’s area and a pathway connecting Broca’s area with the motor cortex. He also added a concept centre to the model; the centre represents diffused, interconnected pathways and memory images, as depicted by Wernicke (Figure 1). The Wernicke–Lichtheim model could account for not only mechanisms of normal language comprehension and production but also the causes of the main forms of aphasic syndromes such as motor and receptive aphasia as well as alexia and agraphia. It has also led to the prediction of new aphasic types such as semantic dementia (loss of word meanings).

With the advancement of techniques for microscopic examination of brain sections (i.e., histology), the associations between brain anatomy and functions became better known. Theodor Meynert pioneered the histological examination of the human brain in 1867 by using a blue dye to observe neuron cells in distinct portions of the cerebral cortex [13]. He observed that different parts of the cerebral cortex had different cell structures and found that sensory input was received at posterior cerebral regions, and motor output was produced at the anterior brain regions [14]. Since then, numerous brain cytoarchitecture maps have been proposed, including Alfred Campbell’s brain map with 14 areas [15], Elliot Smith’s brain map with 50 areas [16], and Korbinian Brodmann’s brain map with 52 areas [17]. Brodmann examined more than 60 mammalian species and divided the cerebral cortex into different areas based on regional variations in cells, labelling each area with a number (from 1 to 52). These regions are now known as Brodmann areas (BAs). Brodmann believed that each area should serve a specific function, and many of them were later found to have a specific role in processing. Although the cortical structure is found to be more complex and heterogeneous than Brodmann suggested, his cytoarchitecture maps remain popular and useful today.

In the last century, neurologists have developed theories of language localisation based on clinical observations and knowledge of the cytoarchitecture of the cerebral cortex. The theories describe typical and atypical language processing mechanisms and offer testable predictions of new aphasic patterns such as conduction aphasia and semantic dementia. Further clinical observations, in turn, provide new data for reinforcing and revising theories. Though many of these neurobiological models have been found to be incorrect and have become obsolete, they still provide a fundamental and schematic understanding of the processing involved in comprehension and production in both auditory and visual modalities. However, interpretations of these findings should be cautious since pathological and healthy brains are different. The language mechanisms deduced from this approach might not reflect the typical brain network. In addition, subjects of those post-mortem brain studies generally had comorbid cognitive or neurological disorders; thus, the brain anomalies observed in them might not be associated with their language deficits.

### fMRI and PET studies on language processing

During the last three decades, our understanding of the functional anatomy of language has been extensively enhanced by neuroimaging techniques. These techniques, including PET and fMRI, provide opportunities to observe brain activity noninvasively in healthy people through careful experimental design. For example, neuroimaging studies of English reading have generally shown that adults recruit left-lateralised brain regions during silent or overt reading, including the inferior frontal, occipitotemporal, and temporoparietal regions [18–21]. The left inferior frontal regions (LIFG), that is, Broca’s area, and the left posterior superior temporal gyrus (LpSTG), that is, Wernicke’s area, serve speech and reading functions. The LIFG, comprising BAs 44, 45, and 47, is involved in multiple functions, including articulatory recoding [22–24], syntactic functions and semantic functions [25], as well as executive functions such as inhibitory control [26–27] and response selection [28]. The LpSTG (or BA 22) is involved in phonemic and rule-based analysis [29–30]. The left angular gyrus (BA 39), the region believed to be a centre for visual word images [31–33], is involved in cross-modal tasks such as visual rhyming and auditory spelling tasks [34–35]. The occipitotemporal region, covering the visual word form area (VWFA), is more activated for written units (either words or letters) than line drawings matching in visual complexity [36–37]. This region is hypothesised to be specialised for orthographic processing [38] and is likely a region that stores visual word images [39], although recent findings have shown that the VWFA is also involved in other cognitive functions, such as attention [40] and nonorthographic visual processing [41]. Though these methods are useful in localising brain functions, they cannot determine the temporal characteristics of signals. Indeed, in recent years, the modular view of brain functions has fallen out of favour, and researchers generally believe that various brain regions function simultaneously and interactively. New analysis methods, such as functional connectivity and dynamic causal modelling, have been used to model dynamic brain networks. To better examine the temporal relation of cortical activities, iEEG would be an ideal method.

### Intracranial studies on aphasia

#### Characteristics of intracranial recording

The iEEG technique provides a promising opportunity to validate and extend current findings from other neuroimaging modalities [42]. The iEEG signal can detect neural activity at the millimetre scale and temporal movement at the millisecond scale. As it provides precise spatial and temporal information about cortical activation and interaction, iEEG is a perfect complement to other invasive or non-invasive neuroimaging methods.

There are two forms of iEEG in terms of the implantation of electrodes. One is called electrocorticography (ECoG), which uses subdural grids or strips of electrodes applied to the cortical surface of the brain. The other is called stereotaxic EEG (sEEG), which uses depth electrodes stereotactically implanted in targeted regions deep inside the brain [42]. Accordingly, ECoG can cover a broader range of brain regions on the cortical surface but may not be used to detect signals from deep brain structures. In contrast, sEEG is advantageous for recording signals from deep brain structures, but electrode placement is more restricted for sEEG than for ECoG. These features cause sEEG to be used less frequently in investigations of higher-level cognitive functions such as language processing. Thus, we focus mainly on studies using ECoG in this review.

**Figure 2:**
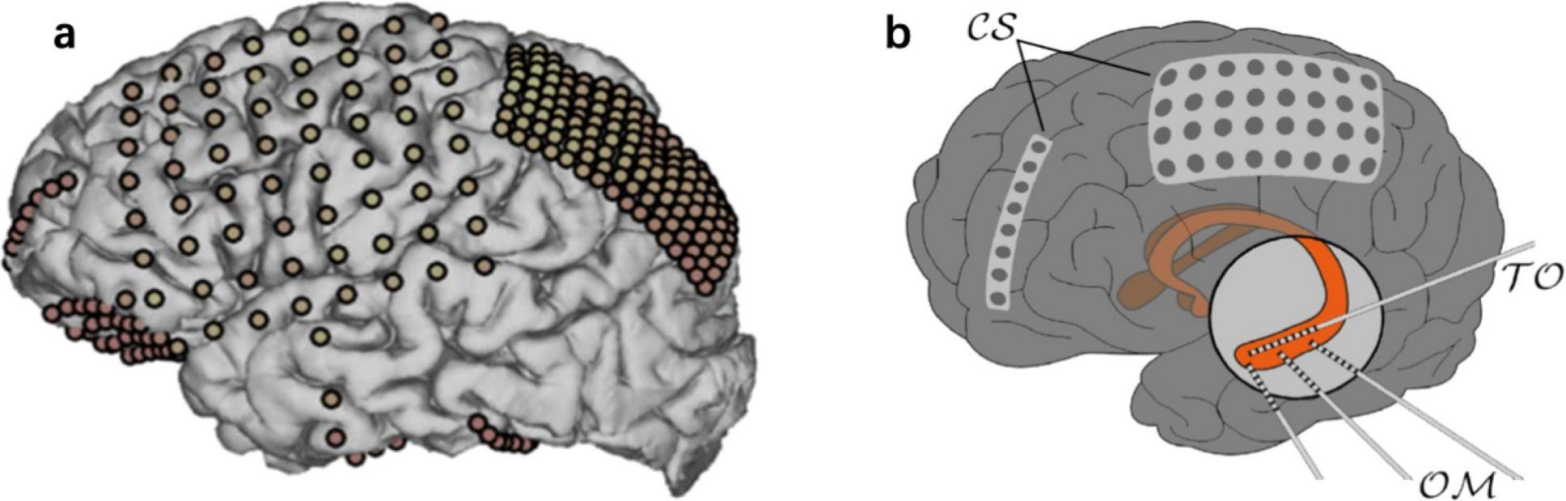
iEEG detects brain activity by implanting electrodes on the cortical surface. (a) Strips and grids of electrodes are used for iEEG recording. Figure 2a was reprinted from [42], Nature Neuroscience, vol. 21, by J. Parvizi; S. Kastner, “Promises and limitations of human intracranial electroencephalography”, Copyright (2018), with permission from Springer Nature. (b) Multicontact sensors could be implanted for iEEG recording. Sensor group cortical surface (CS) are strips and grids of electrodes, and sensor groups trans-occipital (TO) and orthogonal-to-mesial (OM) represent in-depth electrodes. Figure 2b was reprinted from [45], Physiological Measurement, vol. 39, by A. Sanz-Garcia; T. Rings; K. Lehnertz, “Impact of type of intracranial EEG sensors on link strengths of evolving functional brain networks”, article no. 074003, published 13 July 2018, https://doi.org/10.1088/1361-6579/aace94; © Institute of Physics and Engineering in Medicine. Reproduced with permission of IOP Publishing. All rights reserved.

ECoG has been used to investigate the onset zone of seizures in patients with epilepsy for decades [43]. This technique was pioneered by neurologists Wilder Penfield and Herbert Jasper in the early 1950s as part of the Montreal Procedure, a special treatment for severe epilepsy. It remains an important method in epilepsy surgeries for preoperative evaluation and functional cortex mapping [44]. ECoG uses strips and grids of electrodes applied to the cortical surface in either subdural or epidural space (Figure 2a) [42]. For example, in a recent iEEG study (Figure 2b), multicontact sensors were implanted in different brain regions to collect data from patients who suffered from pharmacoresistant focal epilepsies [45]. ECoG offers high spatial resolution. The ECoG grid and strip electrodes can cover a large area of the cortex, and as many as 100–200 electrodes can be used [42]. However, in actual applications, the clinical needs of the patients determine the appropriate grid size and electrode placement. Since most epilepsy patients have left temporal lobe or frontal lobe seizures, electrodes are more commonly implanted in the left hemisphere, covering the medial temporal and frontal cortices. Thus, the parietal, occipital, and deeper brain regions, such as the hippocampus and insula, are less frequently covered [42]. This limitation has significantly restricted the use of ECoG in mapping of widely distributed cognitive networks, such as that for language processing.

Compared to metabolic imaging techniques such as fMRI or PET, ECoG has an excellent temporal resolution at the millisecond scale. ECoG data have a typical sampling rate of 1,000–3,000 Hz. This high temporal resolution offers an opportunity to observe the rapid dynamics of neural activities in precisely localized brain regions. ECoG has a much higher signal-to-noise ratio (SNR) than other modalities, such as fMRI or scalp EEG. The SNR of ECoG is 100 times higher than that of scalp EEG due to the reduction of environmental and physiological noise such as muscle contractions or skin potentials [42,46]. ECoG electrodes are characterised by a circular plate shape, with a diameter of 1.2 to 3 mm, and the centres of two adjacent electrodes are approximately 4 to 10 mm apart [42]. These features enable ECoG electrodes to capture the cell population over a relatively large and diverse range.

The abovementioned characteristics of ECoG have made it a promising recording technique for real-time functional brain mapping. It permits real-time functional mapping of cognitive functions and dynamically images neural pathways between brain regions during cognitive processing, in contrast to fMRI and PET, which offer a static image of functional activity without temporal information. In addition to clinical applications, ECoG provides novel interpretations of functional brain localisation. For instance, a study with five epileptic patients implanted with subdural electrodes in their dominant hemisphere of language demonstrated the potential of using ECoG to temporally and spatially segregate complex cognitive functions within a designated brain region [47]. During ECoG recording, these patients performed different cognitive tasks, which included naming pictures, recognising animal sounds, answering questions, naming visual words, and repeating spoken words. The results showed that ECoG could temporally and spatially segregate the cortical subregions within the left posterior inferior frontal gyrus (LPIFG) regarding different cognitive functions. The visual system located in the occipital lobe would be a good candidate for evaluation by ECoG to study its highly modular but interconnected hierarchical networks [48].

#### iEEG to examine the neuronal representation of reading

As mentioned previously, the ventral occipitotemporal (VOT) region is involved in visual word or orthographic processing [38–39], although whether this region plays specific roles in visual word identification remains a hotly debated topic [49]. To address this issue, Nobre et al. [50] examined ECoG activity at the VOT cortex of 27 patients with epilepsy during sentence reading, word detection, semantic priming, and object perception. The authors found a region in the posterior fusiform gyrus responding equally to word and nonword strings, likely responsible for prelexical letter integration. In contrast, a region in the anterior fusiform gyrus was sensitive to word lexicality (words > nonwords), semantic content, and context. This region could be responsible for concept representation addressable by visual or multimodal images of words. Both regions showed stronger activity to letter strings than objects, implying their specific role in orthographic processing [50–51].

Based on previous neuroimaging studies with epileptic patients, Cohen et al. [51] proposed the visual word form area (VWFA) hypothesis using behavioural, fMRI, and scalp EEG techniques to evaluate five healthy subjects and two patients who had posterior callosal lesions. They observed from the two patients whose VWFA activity, which occurred 150–160 ms after stimulus onset, could be triggered by words presented in the right visual field (RVF) but not the left visual field (LVF). These patients had posterior callosum damage that disconnected their bilateral visual systems. As a result, the information presented to the LVF could not be transmitted to the left hemisphere for orthographic processing. This suggests that the VWFA specific for orthographic processing is confined to the left hemisphere.

**Figure 3:**
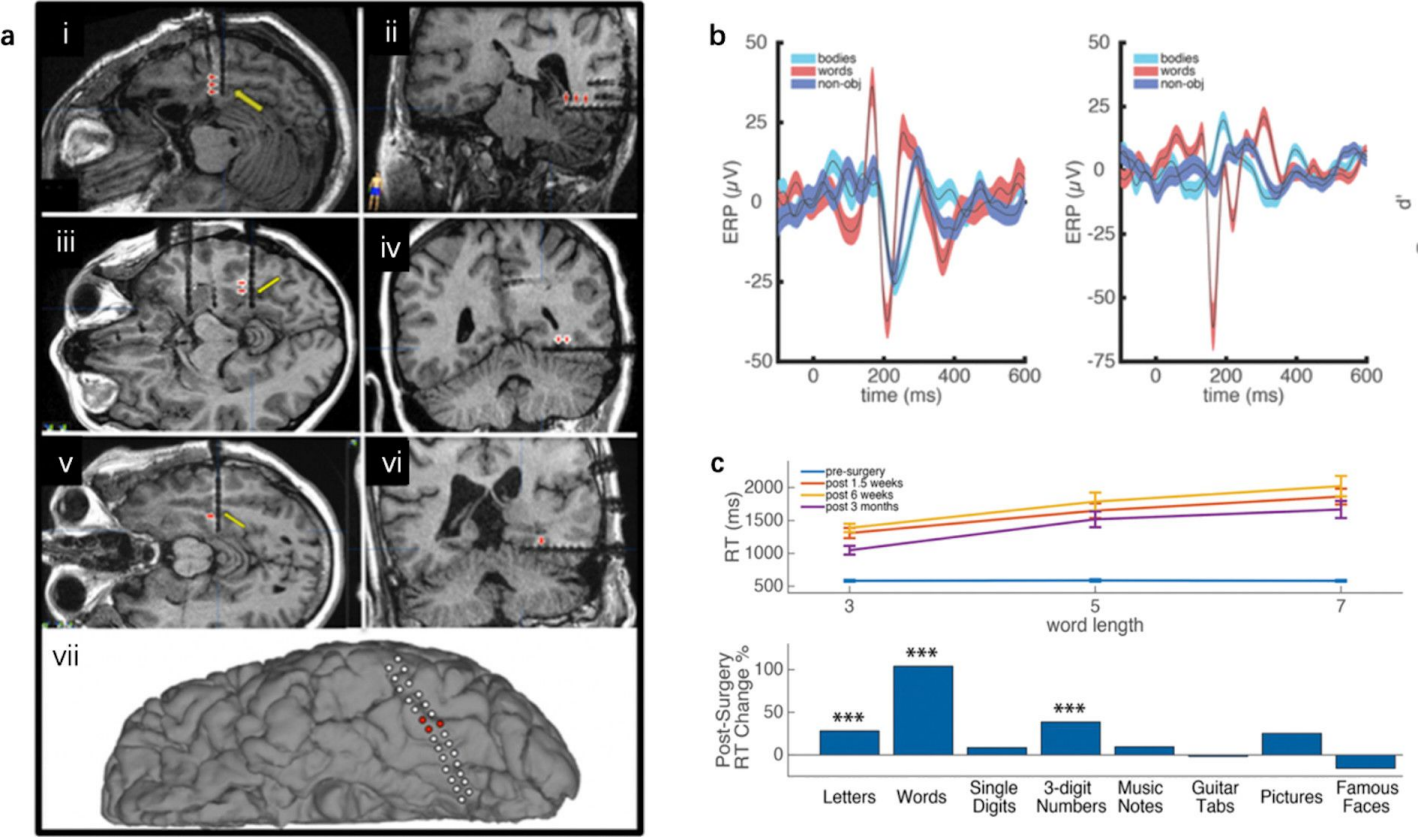
iEEG recording of patients with medically intractable epilepsy. (a) Electrodes were implanted in the ventral temporal cortex of the patients. (b) The signals showed a strong early-stage sensitivity to words at 100–400 ms. (c) The results from the word length and naming task showed that Patient 1 suffered from acquired alexia after surgery. Figure 3 was adapted from [54], Proceedings of the National Academy of Sciences of the United States of America, vol. 113, By E. A. Hirshorn; Y. Li; M. J. Ward; R. M. Richardson; J. A. Fiez; A. S. Ghuman, “Decoding and disrupting left midfusiform gyrus activity during word recognition”, pp 8162–8167, with permission from National Academy of Sciences.

More recent studies have contributed novel findings to support the VWFA hypothesis. Empirical evidence has illustrated that the left mid-fusiform gyrus (lmFG) has a critical role in reading [52–53]. Hirshorn et al. [54] presented iEEG data from four neurological patients with electrodes implanted in their lmFG to demonstrate the involvement of lmFG in word processing (Figure 3a). Four medically intractable epilepsy patients underwent iEEG recordings. The patients viewed three types of visual images (body, word, and phase-scrambled images) and were asked to identify the images that were repeatedly presented. The results showed a strong early-stage sensitivity to word stimuli at 100–400 ms (Figure 3b). Notably, one patient (termed “Patient 1”) underwent surgical removal of tissue around the location of the implanted ventral temporal (VT) electrode. Patient 1 performed multiple naming tasks, including word naming and a set of multiple stimuli pre- and post-surgery at 1.5 weeks, 6 weeks and 3 months. Patient 1 showed reading deficits after the removal of tissues surrounding the VT electrode (Figure 3c). In a follow-up analysis using machine learning methods to examine the patients’ iEEG signals at the VWFA responding to words that differed in the degree of visual similarity, the researchers found that, shortly after stimulus onset (from approximately 100 to 430 ms), discriminating between words that shared no letters would activate the VWFA, but discriminating between those that differed by only one letter would not. However, at a later time window (from approximately 360 to 640 ms), discriminating between any two words would activate the VWFA. This pattern of results suggests that the VWFA processes orthographic information dynamically, shifting from a coarse, lexical-based analysis to fine-grained, sublexical-based processing. Thus, the language network is not only widely distributed across brain regions but is also dynamic. IEEG is a useful technique to capture this dynamic nature of language processing.

Successful reading relies on rapid phonological and semantic encoding of written words [55]. Neuroimaging studies of healthy subjects have detected neural activity in the left superior temporal gyrus and the right superior temporal regions when the subjects are processing phonological information [56]. Semantic processing activates more widely distributed brain regions, including the bilateral temporal and inferior frontal areas [57]. Although most of the neuroimaging findings are acquired using non-invasive techniques such as fMRI or PET, iEEG data can substantiate temporal and spatial characteristics of the non-invasive methods. McDonald et al. [58] conducted a multimodal imaging study to investigate word processing in both healthy people and patients with epilepsy. Twelve healthy participants completed a semantics judgement task while being evaluated with fMRI and MEG, and six patients performed the same task while being evaluated with iEEG. The fMRI and MEG results showed spatial concordance within the bilateral occipitotemporal and medial temporal cortex, the left prefrontal cortex, and the left posterior temporal cortex. IEEG recordings were used to validate the fMRI and MEG measurements. Responses from the abovementioned regions supported the time course of the fMRI and MEG results.

Semantics is a broad concept that covers many different types of information and could be modal or modality-specific (e.g., speech). Thus, there are different forms of semantic processing, such as concreteness judgment (abstract vs concrete words), object categorisation (living vs non-living things), meaning relatedness (table–chair vs table–choir) and meaning association (blue–sky). Also, different semantic processing entails different functional networks [57]. Previous neuroimaging research has investigated semantic processing in language production. However, the results derived from these studies are inconsistent because of the influence of the completed task [59], design of the experiment [60], or the object context [61–62]. Some studies have reported that multiple brain regions, such as occipitotemporal region, posterior parietal cortex, and prefrontal cortex, are involved in semantic processing. Nevertheless, other studies have suggested that the left temporal cortex was the main brain region for semantic processing [63].

**Figure 4:**
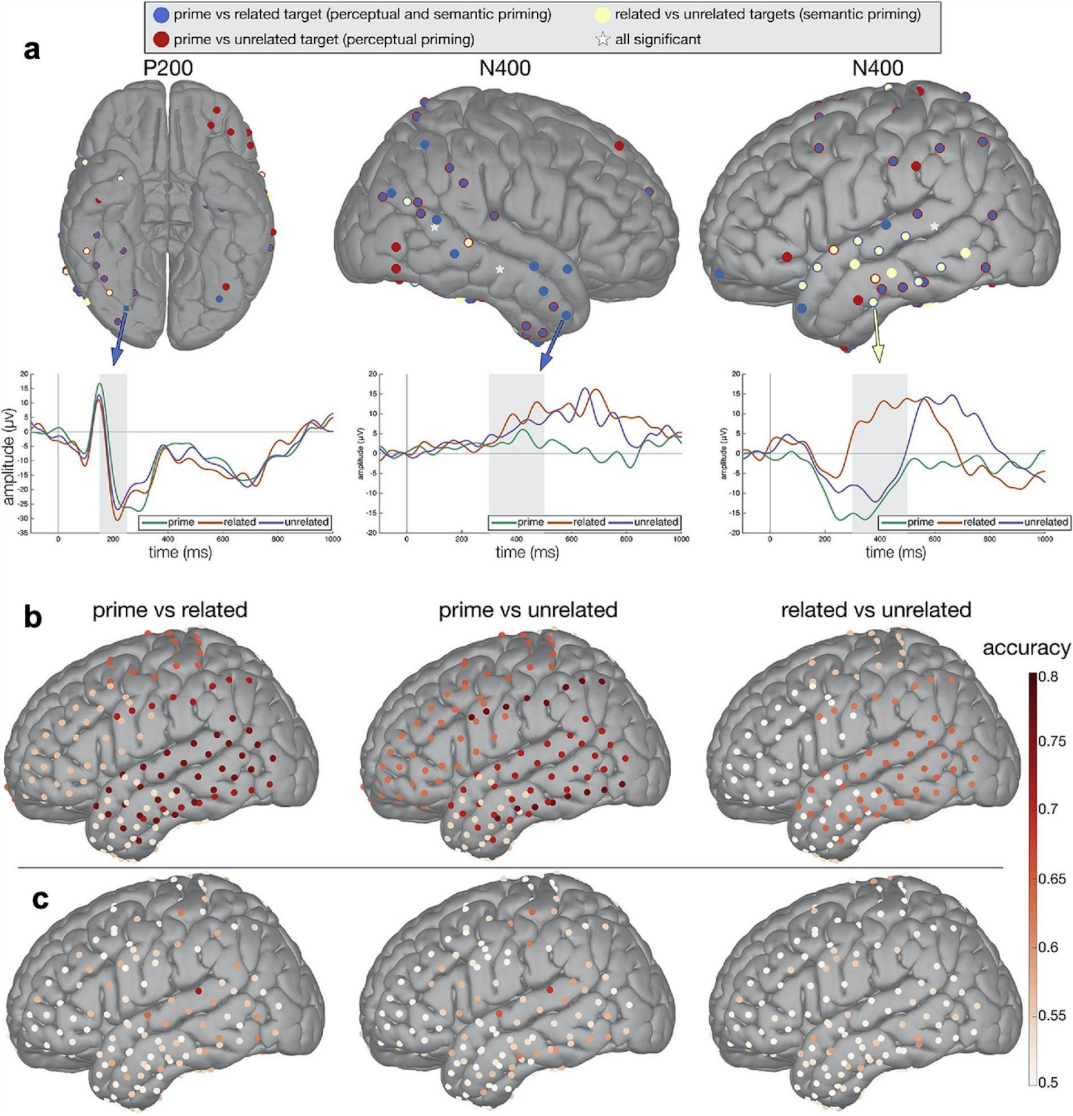
ERP analysis suggests semantic priming centralises in the left temporal cortex, whereas perceptual priming is widespread. (a) ERP analysis suggests the spatial and temporal distribution of semantic and perceptual priming effects. (b) Results of multichannel classification analysis. (c) Results for single channel classification analysis. Figure 4 was reprinted from [64], NeuroImage, vol. 203, by E. Khachatryan; B. Wittevrongel; M. F. Hnazaee; E. Carette; I. Dauwe; A. Meurs; P. Boon; D. van Roost; M. M. Van Hulle, “Semantic and perceptual priming activate partially overlapping brain networks as revealed by direct cortical recordings in humans”, article no. 116204, Copyright (2019), with permission from Elsevier.

As it simultaneously provides insight into the spatial and temporal aspects of semantic processing, iEEG is a promising method to reveal the dynamics of natural semantic processing. Khachatryan et al. [64] recorded neural signals from the scalp and cortex of nine epileptic subjects to study the activation of semantic and perceptual priming (Figure 4). Patients completed a semantic judgment task and also behavioural tasks. Scalp EEG data were collected simultaneously with iEEG data, as intracranial recordings can only be implanted in rare brain regions for clinical purposes. Scalp EEG data have shown that the perceptual priming effect is exhibited in early time windows (N100 and P200). In contrast, the semantic priming effect can only be detected in later (N400 and P600) time windows. For the iEEG data, Khachatryan et al. [64] conducted three analyses, including ERP analysis, time-frequency analysis and classification analysis. As shown in Figure 4, ERP analysis suggested that semantic priming occurs more in the left temporal cortex, whereas the distribution of perceptual priming is rather broad. Time–frequency analysis revealed early participation of the right basal occipitotemporal cortex during object processing and engagement of the left temporal cortex for the semantic and perceptual priming effect. Last, the left temporal cortex showed the highest accuracy of semantic priming in the classification analysis. Thus, the study concluded that semantic and perceptual priming could trigger partially overlapping brain regions during visual object processing.

Phonological processing is crucial for efficient reading comprehension and visual word recognition [65]. Extensive neuroimaging research has revealed the precise location of the cortex and the timing of access to phonological information when recognising visual words. Previous neuroimaging studies have documented brain responses to phonological information in the bilateral superior temporal gyri, left lateralised supramarginal gyrus and left lateralised inferior frontal cortex [66–67]. Furthermore, the relative time course of orthographic, phonological, and semantic activation during word identification remains a focus of debate in reading research [68]. Phonological processing is argued to provide the initial access required for word recognition in natural language processing [69]. With high temporal and spatial resolution, iEEG data provide direct information about how and when phonological processing occurs when recognising visual words. Mainy et al. [70] collected iEEG recordings from ten drug-resistant epileptic patients to reveal the measure of brain activation in the temporal and frontal lobes when processing visual words. To investigate this aspect of phonological processing, patients completed a language decision task involving unreal words while they were evaluated with iEEG. Based on the pattern of gamma-band responses they observed, phonological processing occurred at approximately 400 ms, which was maximally 200 ms after visual analysis. Activations of neuronal populations were observed in the posterior region of Broca’s area, the mid and anterior regions of the superior temporal gyrus, and the lateral prefrontal cortex. The results are compatible with previous neuroimaging findings [71–72] and show a clear time course of orthographic, phonological, and semantic processing.

### The development of intracranial recordings using flexible electronics

Nanotechnology has facilitated the application of neuroimaging by developing brain-compatible neural devices. A novel device fabricated from soft nanomaterials is capable of accurately measuring cortical activity and obtaining in-depth information from target brain regions [73]. These soft nanomaterials are suitable for the invasive iEEG neuroimaging method to detect neurological disorders. Nanomaterial-based flexible intracranial electrodes have been developed also to improve ECoG measurements [74–75]. For instance, conventional ECoG requires the removal of skull bone, which could result in brain damage. Flexible ECoG electrodes have been developed that can reliably attach to the cortex without removing skull bone, improving the recording signals [76].

**Figure 5:**
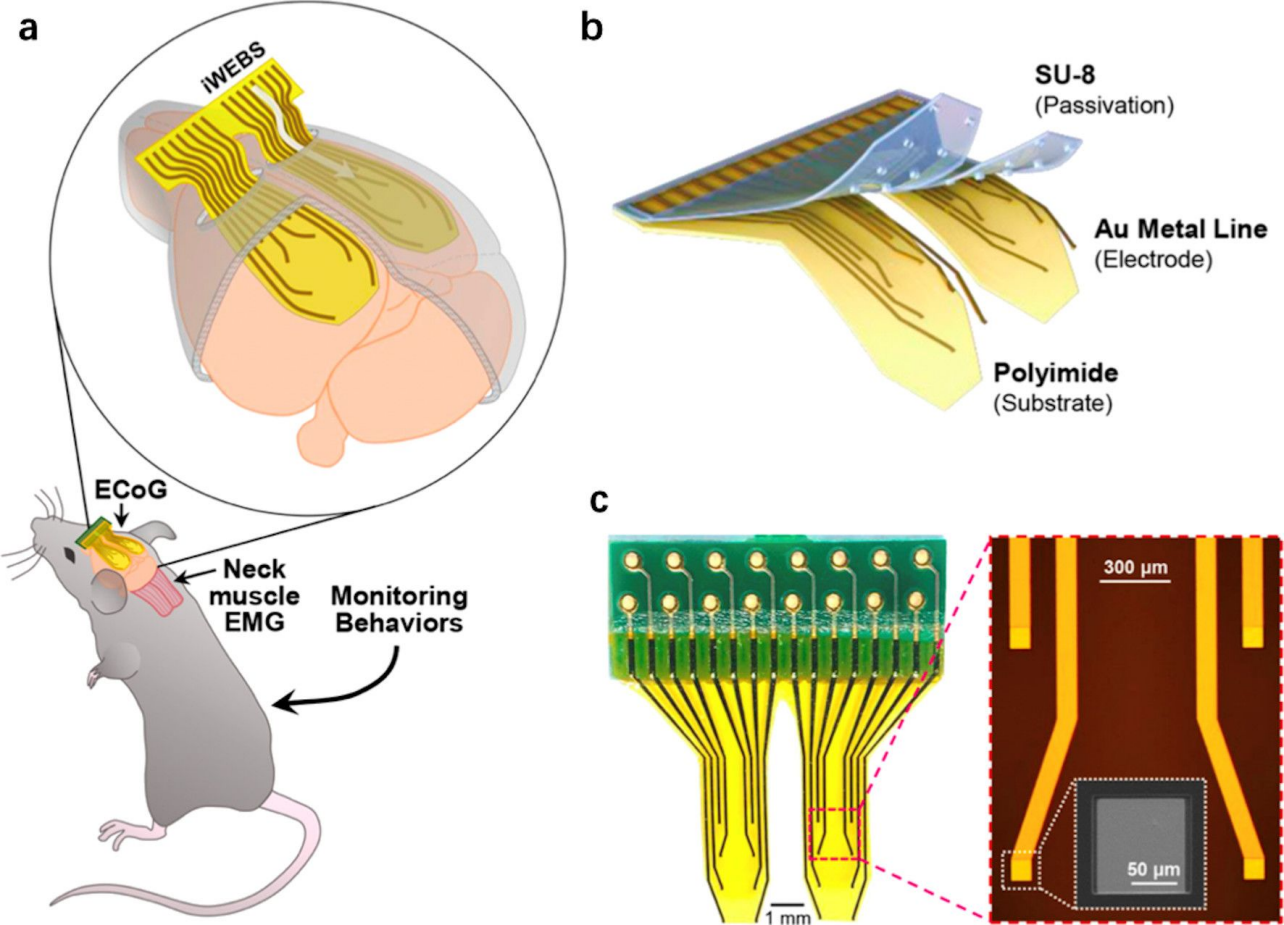
Features of the iWEBS array. (a) Implantation in free-moving mice. (b) Layer components. (c) Structure of recording units. Figure 5 was adapted with permission from [76], Copyright 2016 American Chemical Society.

Based on a flexible microelectrode array, a wrapping electrode array that can be inserted beneath the skull (iWEBS) was fabricated to map cortical connectivity in a wide region, as presented in Figure 5a. The microelectrode array was made of patterned Au wires passivated with SU-8 photoresist on a flexible polyimide (PI) substrate (Figure 5b). The thickness of iWEBSis was only 14.5 µm with 2 µm SU-8 and 12.5 µm PI layers. The bifurcated flap shape was used to achieve good penetration and attachment to the cortical surface and avoid injuring blood vessels on the brain midline (Figure 5c, left). The width of the Au lines was designed to be 100 µm to reach a low impedance value and enhance the signal-to-noise ratio (Figure 5c, right). The real-time signals of cortical activities in different medical and drug-induced epileptic states were precisely measured when the iWEBS array was used in freely moving mice. Combined with optogenetic mapping techniques, long-range cortical interactions were successfully mapped. The application of these flexible electronics in intracranial recording provides insight and opportunity into the study of patients with challenging neural disorders, including aphasia.

Other than insertable devices, researchers have been developing ultrathin intracranial electrodes that could reduce mechanical brain damage. Moreover, some electronics have been tested in animal models [77–81]. Although the human brain is exceedingly distinct from the mouse brain, given the larger size and more complex network, it is possible to replicate the abovementioned techniques in humans with technical development.

Moreover, the highly flexible nature of novel electronics allows for the observation of interactions between distant cortical sites. As discussed above, the disconnection of brain regions may also result in aphasia. Compared to conventional intracranial electrodes, novel electronics such as multichannel ECoG microelectrode arrays provide larger coverage of the brain with exceptional thinness and considerable safety improvements [82]. With the facilitation of nanotechnology, it is possible to obtain a precise picture of the brain network of reading.

## Conclusion

The iEEG technique is a tool that can be used to investigate the neural mechanisms underlying the reading process. Compared to other neuroimaging methods, such as fMRI or PET, iEEG has the advantage of high temporal and spatial resolution. Therefore, iEEG recording provides reliable anatomical precision with more accurate signals. iEEG is especially crucial for understanding the neural mechanism of reading since real-time recording is important for such sophisticated cognitive function. In addition, iEEG recording provides valuable validation to neuroimaging research of reading processing. Although numerous efforts employing multiple neuroimaging methods have been devoted to the elucidation of the anatomical pathway of reading, some results tend to be inconsistent.

Critical limitations of iEEG must be considered when adopting this method. As discussed above, successful reading relies on complex neural processing, including orthographic, semantic and phonological processing. To examine the neurological pathway of reading at different linguistic levels, an explicit experimental design is required. However, iEEG electrodes can only be implanted for clinical purposes. Restricted by configuration, location, and duration of implantation, language tasks must be concise and brief; such tasks may fail to provide sufficient information to address the issue. The lack of data is another limitation for current iEEG studies. The implantation of electrodes is suitable for a small portion of brain-damaged patients with language deficits. Among these patients, those with severe brain damage cannot complete behavioural tasks that are correlated with neuroimaging data. For epileptic patients, only a few will receive neurosurgical treatment that entails brain ablation and implantation with ECoG electrodes. Some epileptic patients are able to partake in language experiments during surgery and participate in behavioural tasks before or after surgery. Fortunately, clinical institutions, such as the US National Institutes of Health and National Science Foundation, have made efforts to build a common platform that encourages researchers to share data across laboratories. A larger number of participants will be recruited to perform identical tasks, thereby illustrating the research question more thoroughly. Broader usage of iEEG is also limited by technique issues. For instance, an adequate number of electrodes should be implanted to accurately detect neural activity [83]. Nevertheless, conventional electrodes are usually 5–10 mm apart; this spacing does not allow information to be read precisely. Furthermore, due to the limitation of the implanted electrodes, iEEG can only be applied to a small portion of the brain with a focus on certain regions. However, a growing number of neuroimaging studies have shown that multiple brain regions far beyond Broca’s area and Wernicke’s area are responsible for language processing. Thus, advanced electrodes that are safe and reliably cover a larger range of the brain are required.

As a continuously evolving field, nanotechnology presents a promising strategy to overcome the current technical limitations of the iEEG method. Flexible electronics have been generated that can be invasively implanted in the brain to collect recordings and simulations of neural activities with high quality. For instance, new models are advanced with significantly reduced thickness, which enables reliable contact with the cortex surface. Thus, more accurate recordings can be acquired. Furthermore, long-term implantation has been used in nonhuman primates or rats that presented steady neural decoding for a long time [84]. The development allows for the observation of brain activity in freely moving animals. Researchers are able to investigate the correlation between behaviour and neural networks. The adaption of nanomaterial-based flexible electronics in iEEG recordings offers a great opportunity to overcome current research limitations and reach new milestones in the future.

Notably, most of the products have been tested in animal models at present. Although animal models have provided fundamental descriptions of neural mechanisms in most sensory and cognitive domains, language, particularly reading, is a more sophisticated brain function exclusive to humans. The adaption of novel flexible electrodes that are specific to the human brain is challenged by the distinct differences between the human and animal brains. For instance, the human brain is significantly larger, has more surface area, contains more neurons and is structurally more complex than that of mice. It is unclear whether flexible electrodes can be expanded to cover a larger cortical surface to collect stable and high-quality neural signals. It is also unclear whether enlarged novel electrodes would release excessive heat and cause damage to the brain, especially during long-term implantation. Further investigations are required to design electronics that are compatible with the human brain for intracranial recording. Fortunately, with the advancement of brain imaging technology, it is highly possible to gain a more precise understanding of the neuronal mechanism of language processing. Sustained research progress in neuroimaging and nanomaterials will facilitate further investigation of brain functions. Ultimately, it is only a matter of time before the gap between research and clinical application is closed.
